# Influence of Maternal Obesity and Gestational Weight Gain on Maternal and Foetal Lipid Profile

**DOI:** 10.3390/nu8060368

**Published:** 2016-06-15

**Authors:** Giulia Cinelli, Marta Fabrizi, Lucilla Ravà, Marta Ciofi degli Atti, Pamela Vernocchi, Cristina Vallone, Emanuela Pietrantoni, Rosalba Lanciotti, Fabrizio Signore, Melania Manco

**Affiliations:** 1 Research Unit for Multifactorial Disease, Bambino Gesù Children’s Hospital, IRCCS (Istituto di Ricovero e Cura a Carattere Scientifico), viale di San Paolo 15, Rome 00146, Italy; giulia.cinelli@opbg.net (G.C.); marta.fabrizi@opbg.net (M.F.); 2 Clinical Epidemiology, Bambino Gesù Children’s Hospital, IRCCS (Istituto di Ricovero e Cura a Carattere Scientifico), P.zza S. Onofrio 4, Rome 00165, Italy; lucilla.rava@opbg.net (L.R.); marta.ciofidegliatti@opbg.net (M.C.d.A.); 3 Unit of Human Microbiome, Genetic and Rare Diseases Area, Bambino Gesù Children’s Hospital, IRCCS (Istituto di Ricovero e Cura a Carattere Scientifico), viale di San Paolo 15, Rome 00146, Italy; pamela.vernocchi@opbg.net; 4 Department of Obstetrics and Gyneacology, San Camillo Hospital, Circonvallazione Gianicolense 87, Rome 00152, Italy; cristina.vallone@libero.it (C.V.); anguela@libero.it (E.P.); fabry64@me.com (F.S.); 5 Centro Interdipartimentale di Ricerca Industriale Agroalimentare, Università degli Studi di Bologna, Piazza Goidanich 60, Cesena 47521, Italy; rosalba.lanciotti@unibo.it; 6 Dipartimento di Scienze e Tecnologie Agro-alimentari, Università degli Studi di Bologna, Piazza Goidanich 60, Cesena 47521, Italy

**Keywords:** pregnancy, fatty acids, biomagnification, bioattenuation, gestational diabetes, gestational weight gain, obesity

## Abstract

Fatty acids (FAs) are fundamental for a foetus’s growth, serving as an energy source, structural constituents of cellular membranes and precursors of bioactive molecules, as well as being essential for cell signalling. Long-chain polyunsaturated FAs (LC-PUFAs) are pivotal in brain and visual development. It is of interest to investigate whether and how specific pregnancy conditions, which alter fatty acid metabolism (excessive pre-pregnancy body mass index (BMI) or gestational weight gain (GWG)), affect lipid supply to the foetus. For this purpose, we evaluated the erythrocyte FAs of mothers and offspring (cord-blood) at birth, in relation to pre-pregnancy BMI and GWG. A total of 435 mothers and their offspring (237 males, 51%) were included in the study. Distribution of linoleic acid (LA) and α-linolenic acid (ALA), and their metabolites, arachidonic acid, dihomogamma linoleic (DGLA) and ecosapentanoic acid, was significantly different in maternal and foetal erythrocytes. Pre-pregnancy BMI was significantly associated with maternal percentage of MUFAs (Coeff: −0.112; *p* = 0.021), LA (Coeff: −0.033; *p* = 0.044) and DHA (Coeff. = 0.055; *p* = 0.0016); inadequate GWG with DPA (Coeff: 0.637; *p* = 0.001); excessive GWG with docosaexahenoic acid (DHA) (Coeff. = −0.714; *p* = 0.004). Moreover, pre-pregnancy BMI was associated with foetus percentage of PUFAs (Coeff: −0.172; *p* = 0.009), omega 6 (Coeff: −0.098; *p* = 0.015) and DHA (Coeff: −0.0285; *p* = 0.036), even after adjusting for maternal lipids. Our findings show that maternal GWG affects maternal but not foetal lipid profile, differently from pre-pregnancy BMI, which influences both.

## 1. Introduction

Maternal diet influences foetus growth and is pivotal for the delivery of healthy, full-term newborns [1]. Maternal metabolism and body composition change through the pregnancy to assure adequate supply of nutrients to the developing foetus. The first trimester is considered an anabolic period that serves to promote maternal fat storage, while the third trimester is deemed to be a catabolic status that favours nutrient supply to the foetus [1].

During pregnancy, fatty acids (FAs) are required (i) as an energy source; (ii) to carry out structural functions (conformation, fluidity and permeability of cellular membranes); and (iii) to act as cellular signalling molecules or precursors of bioactive compounds (prostaglandins, leukotrienes, thromboxanes). These last two functions are provided in particular by essential fatty acids (EFAs), linoleic acid (18:2 *n*-6, LA) and α-linoleic acid (18:3 *n*-3, ALA), and their long-chain metabolites (long-chain polyunsaturated fatty acids, LC-PUFAs): dihomogamma linoleic acid (18:3 *n*-6, DGLA), arachidonic acid (20:4 *n*-6, AA), eicosapentaenoic acid (22:5 *n*-3, EPA) and docosaexahenoic acid (22:6 *n*-3, DHA) [2].

AA, DHA, and EPA are the main components of neuronal membrane phospholipids. They are essential for visual and cognitive development [3].

All the FAs are transported across the placenta from the maternal side to the foetus by specific fatty acid binding/transport proteins [4]. This mechanism is specially effective for all the LC-PUFAs [5,6], particularly for DHA. In physiological conditions, the phenomenon of “bioattenuation” protects the foetus from the excessive passage of maternal DHA through the placenta. Conversely, the “biomagnification” occurs when the DHA percentage in foetal erythrocytes is higher than in mothers [7] and might actually reflect the maternal reduced DHA status [8]. The efficiency of the LC-PUFA transport system has been found to be impaired in maternal dysmetabolic conditions, such as obesity, potentially leading to an altered foetal lipid profile [9]. The Generation R study found obesity and excessive gestational weight gain (GWG) to affect maternal FA plasma profile but it did not investigate the effect on the foetal lipid profile [10].

The main aim of this population study was to evaluate the nutritional FA status, measured in erythrocyte membrane phospholipids, in a large cohort of Italian pregnant women and their offspring. In particular, we aimed (i) to compare maternal and foetal fatty acids profile at birth; and (ii) to explore the effect of pre-pregnancy body mass index (BMI) and GWG on both maternal and foetal lipid profile.

## 2. Materials and Methods

### 2.1. Subjects and Study Design

The “Feeding Low-Grade Inflammation and Insulin Resistance of the Foetus” study is a population study of 1000 mother-infant pairs with the primary aim of evaluating the association at birth between maternal erythrocyte concentrations of FAs and the child’s insulin resistance and low-grade inflammation. Healthy pregnant women (aged 18–45 years) were enrolled, at the San Camillo Forlanini Hospital (SCH) in Rome, from February 2013 until June 2015 and followed up from the 1st trimester of pregnancy to childbirth with monitoring of lifestyle, blood testing, and ultrasonography, according to the guidelines of the Italian Society for Gynaecology and Obstetrics [11].

Inclusion criteria were being at week 7–10 of gestation, folic acid supplementation from week 7, singleton pregnancy, no alcohol or medications, no systemic, chronic, or autoimmune disease, no previous diagnosis of Gestational Diabetes Mellitus (GDM) or miscarriage, no conception through ovulation induction or *in vitro* fertilization, planned delivery at SCH-Unit, maternal and foetal fatty acids erythrocyte profiles.

The “Feeding” study was approved by the Ethical Committees of the “Ospedale Pediatrico Bambino Gesù” (OPBG) and the SCH, in full agreement with the national and international regulations and the Declaration of Helsinki (2000). All the participants signed an informed consent.

### 2.2. Anthropometrics and Clinical Evaluation

Mothers’ body weight and height were measured at the enrolment following international guidelines [12]. Repeated measures of body weight were performed during pregnancy until childbirth. Pre-pregnancy body weight was used as reference and, in case of incongruities, the patient’s general practitioner was consulted. Pre-pregnancy BMI was calculated as kg/m^2^ and classified according to the World Health Organization (WHO) [13]. GDM was diagnosed according to the American Diabetes Association’s (ADA’s) Standards of Care [14].

GWG was calculated by subtracting the pre-pregnancy weight to the weight reached at time of delivery. According to the Institute of Medicine (IOM) guidelines, we defined adequate gestational weight gain in relation to pre-pregnancy BMI (12.5–18 kg in underweight; 11.5–16.0 kg in normal weight; 7.0–11.5 kg in overweight and 5.0–9.0 kg in obesity). Otherwise, it was defined as inadequate or excessive if weight gain was below or exceeded values recommended for pre-pregnancy BMI classes, respectively.

At each trimester, women underwent a 40-minute interview with a nutritionist (GC) to estimate food consumption frequencies and received recommendations for healthy eating habits.

The following sociodemographic and anthropometric data for both the parents were collected to estimate socioeconomic status (SES); race; level of education; profession; smoking and parity.

Newborns’ anthropometrics (body weight, BW; body length, BL and head circumference, HC) were evaluated at birth according to standardized procedures [15]. Standard deviation scores (SDS) for infant weight and height was calculated following the Italian INeS (Italian Neonatal Study) Chart [15].

### 2.3. Samples Collection

Maternal blood samples were withdrawn at fasting, 12–24 h before giving birth, during the pre-partum foetal monitoring. Cord-blood samples (2.5 mL) were collected at birth by venipuncture from the placental portion of the umbilical cord immediately after clamping.

Blood was placed in ethylenediaminetetraacetic acid (EDTA) tubes. Erythrocyte membranes were isolated within 2 h after collection: plasma was separated by centrifugation (980 rpm, 18 min); whereas erythrocytes were added with acid citrate dextrose, washed with distilled water (10:1) and centrifuged (4000 rpm, 5 min) four times. Erythrocytes were frozen immediately at −80 °C and stored until lipid extraction.

### 2.4. Fatty Acid Analysis

#### 2.4.1. Lipid Extraction from Erythrocyte Membranes

Erythrocytes were resuspended into hexane/methanol (1:3) solution and homogenized by vortexing for 1 min. Tubes were stored at 4 °C for 5 min and then 400 µL of acetyl chloride were added, by a careful handling, and incubated at 100 °C for 1 h. After incubation, the tubes were cooled down to 4 °C for 30 min, then 3 mL of K2CO3 (12%) were added.

At the end of CO_2_ production, the tubes were homogenized by manual inversion for 1 min. The mixture was then centrifuged at 3500 rpm for 5 min. The supernatant was transferred in gas-chromatography vials and the solvent was removed by using the GeneVac EZ-2 Plus evaporator (GeneVac, New York, NY, USA). The methyl-C11 (2 mg/mL) was added as internal standard.

#### 2.4.2. Gas Chromatography Analysis of Fatty Acid Methyl Esters

Analyses of fatty acid methyl esters (FAME) were performed by a fast gas-chromatography/flame-ionization detector 2010 Plus with an autosampler AOC-20i (Shimadzu, Kyoto, Japan), equipped with a fused silica BPX70 capillary column (10 m × 0.1 mm I.D, 0.2 µm film thickness; SGE, Melbourne, Australia). Split injector (100:1) and flame-ionization detector system were operating at 250 °C.

The oven temperature programming at injection was 50 °C isothermal for 0.2 min, increased to 175 °C at 120 °C/min, then increased to 220 °C at 20 °C/min and finally to 250 °C at 50 °C/min.

The carrier gas (H2) flow was maintained at 0.8 mL/min and the volume injected was 0.30 µL. The method was optimized in house according to Destillas *et al.* [16].

The chromatograms were integrated and identified by comparing the retention times and the peak area with those of a commercial lipid standard of 52 fatty acids (GLC 463 Nuchek; Elysian, MN, USA) and a conjugated linoleic acids mixture (UC-59M Nuchek; Elysian, MN, USA). Quantitative data were obtained by interpolation of the relative areas *vs.* internal standard (Methyl-C11) area. Data are shown as FAME concentration (ng/mL) or percentage (% of total FAME).

### 2.5. Statistical Analysis

Data are represented as number and percentage in parentheses (%) for categorical variables, or median and interquartile range (IRQ) for continuous variables. To evaluate differences and correlations between maternal and foetal fatty acid compositions, Wilkoxon Signed-Rank test was performed and Spearman correlation coefficient was calculated on concentration (ng/mL) and percentage of total FAs.

Quantile regression analyses were conducted on median percentage of total FAs to investigate the association between pre-pregnancy BMI and classes of GWG and maternal/foetal erythrocyte lipid profile. Multivariate quantile regression analyses, adjusted for maternal characteristics (GWG, pre-pregnancy BMI, smoking, age, educational level, offspring sex, gestational age, parity) were conducted on median maternal lipid profile. Multivariate quantile regressions, adjusted also for maternal lipid profile, were run on foetal lipid profile, in order to estimate the effect of these conditions on foetal erythrocyte fatty acid composition. All covariates were included in the multivariate models and the final one was determined through a backward approach.

Statistical analysis was performed through Stata 13.1 software (StataCorp, 4905 Lakeway Drive, College Station, TX, USA).

## 3. Result

### 3.1. Subjects

From the initial cohort of 1000 pregnant women enrolled, 144 (14.4%) mothers withdrew from the study (6 genetic diagnoses, 12 childbirth complications not allowing blood collection, 24 personal reasons, 7 miscarriages, and 95 deliveries in different hospitals). No difference was found in age, anthropometrics and SES of women who participated or withdrew the study (data not reported).

Data on lipid profile were missed in 396 (39.6%) pairs. A complete data set of fatty acid profile was available for 460 mother-infant pairs (46%); 449 (97.6%) Caucasians. Two (0.4%) women had T1D and 6 (1.3%) T2D at time of enrolment, while 17 (3.7%) pregnant women were diagnosed with GMD. The data of 25 (5.4%) women were not considered for the present study and a final sample of 435 mother-infant pairs was used for the analysis.

Table 1 shows maternal and foetal characteristics of the sample.

### 3.2. Fatty Acid Profile in Maternal and Foetal Erythrocyte Membranes

#### 3.2.1. Saturated Fatty Acids

Table 2 shows maternal and foetal FA composition of erythrocytes, expressed in percentage (% of total FAs). Concentrations are expressed in Table S1. Total saturated fatty acids (SFAs) were reported to be the most represented class, both in maternal and foetal erythrocytes. A significant difference in total SFA between mothers and newborns was detected (*p* < 0.0001). For example, stearic acid (18:0) was significantly higher in the foetus (*p* < 0.0001), while we found arachidic acid (20:0, *p* < 0.0001) and behenic acid (22:0, *p* < 0.0001) to be lower and higher in maternal than in foetal erythrocytes, respectively.

The SFA/UFA ratio appeared to be significantly higher in foetal erythrocytes (*p* < 0.0001).

#### 3.2.2. Unsaturated Fatty Acids

Total MUFAs, PUFAs, *n*-3 and *n*-6 percentage were found to be significantly higher in maternal than cord-blood erythrocytes. In particular, palmitoleic acid (*cis* 16:1 *n*-7; *p* < 0.0001), oleic acid (18:1 *n*-9; *p* < 0.0001) and trans-vaccenic acid (*trans* 18:1 *n*-7; *p* < 0.0001) were differently represented in maternal *vs.* foetal erythrocytes. *n*-6/*n*-3 ratio appeared to be significantly higher in maternal erythrocytes (*p* < 0.0001).

#### 3.2.3. Essential Fatty Acids (EFAs) and Long-Chain Unsaturated Fatty Acids (LC-PUFAs)

Distribution of EFAs was statistically different in maternal erythrocytes compared to cord blood (LA: *p* < 0.0001; ALA: *p* < 0.0001). AA was the most represented *n*-6 LC-PUFA, whereas DHA was the most present *n*-3 LC-PUFA, in both maternal and foetal total erythrocyte lipids. Among the *n*-6 LC-PUFAs, AA and DGLA were significantly higher in cord-blood erythrocytes (AA: *p* < 0.0001; DGLA: *p* < 0.0001). Among the *n*-3 LC-PUFAs, EPA and DPA were significantly higher in maternal erythrocytes (*p* < 0.0001 for both).

A general positive correlation was detected between maternal and foetal FA percentage.

**Figure 1:**
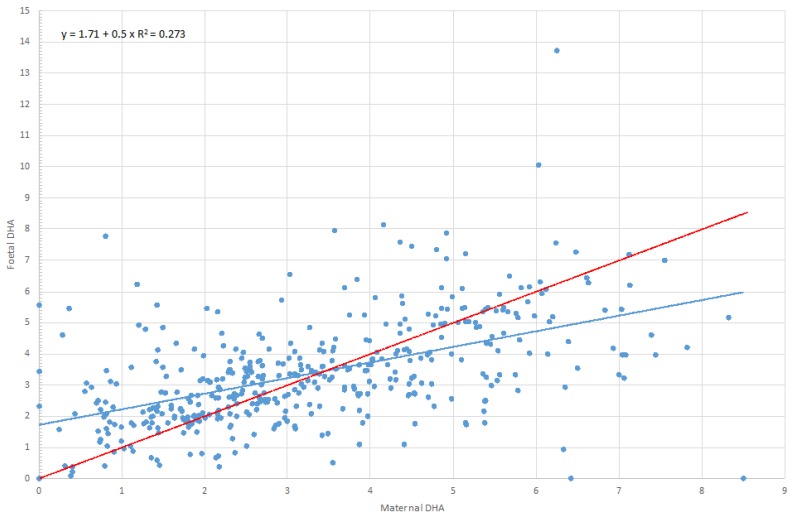
Relationship between maternal and foetal DHA percentage at delivery. Dashed line indicates *y* = *x* for “equal maternal and foetal DHA%”. Continuous line indicates the relationship between maternal and foetal DHA%. At delivery, maternal DHA% equals foetal DHA at 3.4%.

Since no significant difference was found in DHA percentage, using the equation shown in Figure 1, at *y* = *x*, we calculated that in our population maternal DHA levels of ≤3.4% corresponded with higher infant DHA%, whereas infant DHA was lower than maternal DHA at >3.4%. Mothers with a percentage of DHA ≤3.4% were 247 (56.8%) and those with a percentage of DHA >3.4% were 188 (43.2%). In the first group, we observed a significant positive trend for DHA transfer from mothers to foetus (maternal DHA = 2.2 (IQR: 1.4–2.7); foetal DHA = 2.6 (IQR: 1.9–3.4); *p* < 0.0001); the opposite was observed in the second group (maternal DHA = 4.7 (IQR: 4.1–5.6); foetal DHA = 4.1 (IQR: 3.2–5.3); *p* < 0.0001) (Figure S1).

### 3.3. Association between Maternal Characteristics and Maternal and Foetal Lipid Profile

#### Pre-Pregnancy Body Mass Index (BMI) and Gestational Weight Gain (GWG)

Quantile regressions were conducted to explore the possible association between pre-pregnancy BMI or classes of GWG and the maternal/foetal erythrocyte lipid profile (expressed in percentage). Tables S2 and S3 show statistically significant results for univariate quantile regression for maternal and foetal FAs percentages, respectively.

Multivariate quantile regressions demonstrated an association between pre-pregnancy BMI and maternal MUFAs (Coeff.: −0.112; *p* = 0.021), LA (Coeff.: −0.033; *p* = 0.044) and DHA (Coeff.: 0.055; *p* = 0.016); between inadequate GWG and DPA (Coeff.: 0.637; *p* = 0.001); between excessive GWG and DHA (Coeff.: −0.714, *p* = 0.004) (Table 3).

Pre-pregnancy BMI was also associated with PUFAs (Coeff.: −0.172; *p* = 0.009), *n*-6 (Coeff.: −0.098, *p* = 0.015) and DHA (Coeff.: −0.029; *p* = 0.036) on foetal erythrocyte membrane (Table 4).

## 4. Discussion

Findings of the present study demonstrated first that pre-pregnancy BMI affects maternal and foetal erythrocyte lipid profile, while GWG seems to influence only maternal profile. Pre-pregnancy BMI was negatively associated with maternal LA and MUFAs and positively with DHA. In foetal erythrocytes, pre-pregnancy BMI was inversely associated with PUFAs, DHA and n-6 FAs. Inadequate GWG was, conversely, correlated to an increased maternal DPA while the excessive GWG WAS associated with a decreased maternal DHA, albeit with no effect on foetus lipid profile in both cases.

Maternal lipid profile likely changes during the pregnancy to adapt to the developing foetus demand [1]. By the end of full-term pregnancies (37–41 weeks), we found that the percentage of PUFAs, particularly *n*-3 FAs and DHA, significantly increased with the gestational age (Table 2). The Generation R study is the largest cohort study that investigated the influence of pre-pregnancy BMI and GWG in 5636 women on maternal lipid profile, but at mid-pregnancy and on plasmatic concentration of fatty acids. Hence, our findings are only partly comparable to those of the Generation R study, which found a higher pre-pregnancy BMI associated with total SFAs and n-6 PUFAs concentrations in women’s plasma and a decrease of LA [10]. In keeping with the Generation R findings [10], we observed a lower percentage of LA at the end of pregnancy in mothers whose pre-pregnancy BMI was higher. In addition, we found that pre-pregnancy BMI was positively associated with DHA percentage in mothers and negatively in foetuses. It is unlikely that the former association reflects a higher fish intake in obese mothers with respect to normal weight since most of them consumed less than three servings per week. On the contrary, it might suggest an impaired transfer of DHA from mother to foetus, which has been previously hypothesised in obese mothers [17]. Obesity, indeed, has been associated with an impaired expression of FAs carriers on the placenta membranes [18]. The clinical impact of the latter association is minimal since it means 0.03% difference in foetuses’ DHA percentage per maternal pre-pregnancy BMI point. As to excessive GWG, the Generation R study found an association with plasmatic concentrations of SFAs, MUFAs and *n*-6 PUFAs in mothers [10].

Very few and only small-sample studies compared maternal and foetal lipid profile on erythrocyte membranes [19,20,21,22]. By comparing pairs’ profiles, it seems that AA and DGLA are preferentially transferred across the placenta with respect to LA, ALA *n*-3 EPA and DPA [19,20,21,22]. In our cohort, the distribution of LA and ALA in maternal and cord-blood erythrocytes, compared to AA, confirms the preferential LC-PUFAs transfer to the foetus [23]. In a large proportion of mothers (55%), we observed a very low percentage of ALA but higher DHA content, supporting the notion of favoured ALA conversion to DHA at the end of pregnancy to supply the foetus’s demand. DHA is essential for the foetal neurodevelopment in late pregnancy [3]. Even if foetal tissues are able to convert the precursor ALA into DHA, foetal ability is relatively low [24], hence placental transfer from the mother is the major source of DHA [25]. It has been supposed that in the case of excessive supply of DHA from the mother to the foetus, the passage of DHA through the placenta is inhibited in a process known as “bioattenuation”, possibly to prevent DHA competition with AA in infant organs [8]. Conversely, whenever the maternal percentage of DHA is reduced (in our population, below 3.4% of the whole FAs content), the placental transport is favoured, resulting in higher foetal DHA (*i.e.*, “biomagnification”) [2,7]. Biomagnification might be confined to populations with low maternal DHA status [8,19]. The percentages of DHA in our sample were comparable, and even lower than the ones detected in populations with low fish consumption [20,26]. Indeed, only ~6% of the women included in our sample reported eating three or more portions of fish per week, whereas 6% stated not eating fish at all. Isolating the 27 women with the highest intake of fish, the percentage of DHA rose up to a mean value of ~5%. In keeping with this hypothesis by further dividing into two subsamples (Figure S1), we found different trends in maternal and foetal percentage of DHA.

Our study, including 435 mother-infant pairs, was the largest one in the literature investigating the erythrocyte fatty acids profile of mothers and their offspring at the end of the pregnancy. Erythrocyte FAs reflect dietary intake in the last 40 days of pregnancy and provide a more accurate estimate of fat consumption than any dietary recall [27]. To our knowledge, it was also the first study exploring the association between pre-pregnancy BMI and GWG and maternal and foetal lipid profile. As a major limitation, however, we recognize the lack of information on the eventual DHA supplementation during the pregnancy.

Furthermore, due to a delay in funding, it was not possible to complete the lipid profile of all the stored samples. A complete dataset of fatty acids in both mother and newborn was available in 460 pairs. There was no difference in anthropometrics, clinical and SES characteristics of pairs whose lipid profile was available with respect to mother-newborn pairs whose profile was not analysed.

In conclusion, our results suggest the obesity status more than excessive weight gain can favour an adverse foetal lipid profile at the end of pregnancy. In the present cohort, the pre-pregnancy BMI affected the foetal lipid profile, being associated with decreased PUFAs, both *n*-6 and DHA, and likely owing to an impaired transfer across the placenta. Even though no association was found between maternal weight gain and foetal lipid profile, caution must be paid to maternal DHA levels and future research is needed in this regard. Further studies are also required to investigate the underlying mechanisms that regulate nutrient sensing across the placenta.

## Figures and Tables

**Table 1:** Maternal and infant characteristics.

Variable		*n* = 435
**Mothers**	Age (years)	33.0 (29.0–37.0) ^1^
	Pre–pregnancy BMI (kg/m^2^)	21.9 (20.0–24.6) ^1^
	Underweight	28 (6.4) ^2^
	Normal weight	309 (71.0) ^2^
	Overweight	65 (15.0) ^2^
	Obese	33 (7.6) ^2^
	GWG	13.0 (10.0–16.0) ^1^
	Inadequate GWG	124 (28.5) ^2^
	Adequate GWG	167 (38.4) ^2^
	Excessive GWG	144 (33.1) ^2^
	Maternal smoking in pregnancy	73 (16.8) ^2^
	Parity	
	0	242 (55.6) ^2^
	1	154 (35.4) ^2^
	≥2	39 (9.0) ^2^
	Gestational age (wk)	39.4 (38.6–40.6) ^1^
	Delivery method	
	Vaginal	271 (62.3) ^2^
	Caesarean	164 (37.7) ^2^
	Education level	
	Primary school	2 (0.5) ^2^
	Secondary school	60 (13.8) ^2^
	High school	209 (48.0) ^2^
	Bachelor degree	164 (37.7) ^2^
**Infant**	Infant sex: male	229 (52.6) ^2^
	Birth weight (g)	3330 (3060–3630) ^1^
	Birth length (cm)	50.0 (49.0–52.0) ^1^
	Cranial circumference (cm)	35.0 (34.0–36.0) ^1^
	SDS weight infant	0.2 (1.2) ^2^
	SDS height infant	0.4 (1.1) ^2^

Data are expressed as the median and interquartile range (IQR) ^1^ or as number and percentage (%) ^2^; BMI, body mass index; GWG, gestational weight gain; SDS, standard deviation score; wk, weeks. Anthropometric measures were taken at study enrolment for mothers, at birth for infants.

**Table 2:** Percentage of FAs in maternal and foetal erythrocytes (*n* = 435).

Fatty Acids ^a^	Mean	Median	25° Perc.	75° Perc.	Mean	Median	25° Perc.	75° Perc.	Correlation Maternal *vs.* Fetal2		
	Maternal				Foetal				*p* ^a^	*r* ^b^	*p* ^b^
**12:0**	1.21	0.87	0.00	2.16	1.23	0.89	0.00	2.27	0.932	0.6	***0.000***
**13:0**	0.34	0.00	0.00	0.43	0.36	0.00	0.00	0.50	0.930	0.6	***0.000***
**14:0**	1.05	0.94	0.60	1.43	1.02	0.88	0.57	1.37	0.247	0.6	***0.000***
**15:0**	0.50	0.46	0.25	0.71	0.49	0.40	0.18	0.65	***0.001***	0.4	***0.000***
**16:0**	20.06	19.75	17.29	22.67	19.41	19.40	16.53	22.23	***0.000***	0.6	***0.000***
**17:0**	0.47	0.47	0.23	0.65	0.46	0.42	0.24	0.56	***0.005***	0.6	***0.000***
**18:0**	13.77	13.41	11.70	15.51	14.15	14.29	12.00	16.15	***0.000***	0.6	***0.000***
**19:0**	0.04	0.00	0.00	0.00	0.02	0.00	0.00	0.00	0.417	0.4	***0.000***
**20:0**	0.36	0.42	0.29	0.50	0.40	0.47	0.27	0.57	***0.000***	0.5	***0.000***
**22:0**	1.27	1.42	1.21	1.62	1.06	1.20	0.95	1.38	***0.000***	0.6	***0.000***
**24:0**	3.88	4.20	3.56	4.75	3.79	4.10	3.41	4.74	0.370	0.5	***0.000***
***cis* 12:1, *n*-1**	0.87	0.44	0.00	1.61	0.87	0.35	0.00	1.67	0.441	0.8	***0.000***
***cis* 14:1, *n*-5**	0.72	0.44	0.00	1.23	0.69	0.40	0.00	1.25	0.160	0.8	***0.000***
***cis* 15:1, *n*-1**	0.35	0.00	0.00	0.28	0.15	0.00	0.00	0.23	***0.008***	0.7	***0.000***
***trans* 16:1, *n*-7**	0.22	0.08	0.00	0.30	0.28	0.00	0.00	0.46	0.739	0.2	***0.000***
***cis* 16:1, *n*-7**	0.58	0.49	0.00	0.87	0.41	0.41	0.00	0.71	***0.000***	0.3	***0.000***
***cis* 17:1, *n*-7**	0.38	0.00	0.00	0.79	0.26	0.00	0.00	0.55	***0.000***	0.7	***0.000***
***trans* 18:1, *n*-9**	0.24	0.00	0.00	0.00	0.24	0.00	0.00	0.00	0.515	0.4	***0.000***
***cis* 18:1, *n*-9**	8.51	9.88	1.53	13.01	5.74	6.79	1.09	8.92	***0.000***	0.7	***0.000***
***trans* 18:1, *n*-7**	3.27	0.00	0.00	7.99	2.66	0.00	0.00	6.94	***0.000***	0.7	***0.000***
***cis* 18:1, *n*-7**	1.00	1.01	0.78	1.22	1.33	1.38	1.09	1.65	***0.000***	0.3	***0.000***
***cis* 19:1, *n*-9**	0.17	0.00	0.00	0.30	0.15	0.00	0.00	0.22	***0.002***	0.6	***0.000***
***cis* 20:1, *n*-15**	0.04	0.00	0.00	0.00	0.04	0.00	0.00	0.00	0.681	0.6	***0.000***
***cis* 20:1, *n*-12**	0.18	0.00	0.00	0.35	0.11	0.08	0.00	0.18	***0.000***	0.6	***0.000***
***cis* 20:1, *n*-9**	0.15	0.00	0.00	0.30	0.10	0.00	0.00	0.17	***0.000***	0.6	***0.000***
***cis* 22:1, *n*-9**	0.08	0.00	0.00	0.14	0.10	0.00	0.00	0.14	0.825	0.5	***0.000***
***cis* 24:1, *n*-9**	4.69	5.31	3.67	6.27	3.20	3.70	1.31	4.43	***0.000***	0.6	***0.000***
**18:2, *n*-6 (LA)**	5.19	5.58	4.49	6.47	2.43	2.32	1.92	2.78	***0.000***	0.2	***0.000***
**18:3, *n*-6**	0.18	0.14	0.00	0.24	0.13	0.10	0.00	0.20	***0.000***	0.5	***0.000***
**18:3, *n*3 (ALA)**	0.08	0.00	0.00	0.14	0.06	0.00	0.00	0.00	***0.000***	0.2	***0.000***
**20:2, *n*-6**	0.21	0.23	0.00	0.35	0.50	0.54	0.29	0.69	***0.000***	−0.1	***0.021***
**20:3, *n*-6 (DGLA)**	1.09	1.17	0.82	1.46	1.42	1.51	1.15	1.87	***0.000***	0.5	***0.000***
**20:4, *n*-6 (AA)**	6.39	7.23	4.28	9.19	7.35	8.54	5.67	10.17	***0.000***	0.5	***0.000***
**20:3, *n*-3**	0.65	0.00	0.00	0.07	0.88	0.00	0.00	0.07	0.510	0.6	***0.000***
**20:5, *n*-3 (EPA)**	0.23	0.22	0.00	0.33	0.18	0.15	0.00	0.25	***0.000***	0.4	***0.000***
***trans* 22:2, *n*-7**	0.54	0.49	0.36	0.74	0.33	0.29	0.00	0.50	***0.000***	0.2	***0.000***
**22:5, *n*-3 (DPA)**	0.70	0.41	0.00	1.05	0.44	0.00	0.00	0.58	***0.000***	0.7	***0.000***
**22:6, *n*-3 (DHA)**	3.30	3.08	1.97	4.48	3.37	3.19	2.21	4.16	0.280	0.6	***0.000***
**Total FAs**	99.77	100.00	100.00	100.00	100.00	100.00	100.00	100.00	0.339	0.0	0.849
**Total SFAs**	42.99	42.46	38.46	47.02	42.37	42.22	37.79	47.50	***0.000***	−0.3	***0.000***
**Total MUFAs**	21.73	21.59	18.74	24.18	16.64	16.24	14.45	18.30	***0.000***	0.4	***0.000***
**Total PUFAs**	19.93	20.75	15.71	24.27	18.75	19.37	15.49	22.31	***0.000***	1.0	***0.000***
**Total *n*-3**	4.95	4.48	2.77	6.06	4.94	4.22	3.04	5.75	***0.000***	0.3	***0.000***
**Total *n*-6**	12.83	13.84	9.81	15.99	11.36	12.66	9.16	14.61	***0.000***	0.4	***0.000***
**SFA/UFA ratio**	1.06	1.01	0.88	1.18	1.24	1.19	1.01	1.37	***0.000***	0.4	***0.000***
***n*-6/*n*-3 ratio**	3.81	3.23	2.20	4.72	3.12	3.21	2.01	4.08	***0.000***	0.6	***0.000***

AA, arachidonic acid; ALA, alpha-linolenic acid; DGLA, dihomo-gamma-linolenic acid; DHA, docosahexaenoic acid; DPA, docosapentaenoic acid; EPA, eicosapentaenoic acid; LA, linoleic acid; FAs, fatty acid methyl ester; MUFAs, monounsaturated fatty acids; Perc., percentiles; PUFAs, polyunsaturated fatty acids; SFAs, saturated fatty acids; UFAs, unsaurated fatty acid; *vs.*, *versus*. Total SFAs include: 12:0. 13:0. 14:0. 15:0. 16:0. 17:0. 18:0. 19:0. 20:0. 22:0. 24:0. Total MUFAs include: *cis* 12:1 *n*-1. 14:1 *n*-5. *cis* 15:1 *n*-1. *trans* 16:1 *n*-7. *cis* 16:1 *n*-7. *cis* 17:1 *n*-7. *trans* 18-1 *n*-9. *cis* 18:1 *n*-9. *trans* 18-1 *n*-7. *cis* 18:1 *n*-7. 18:1 *n*-5. *n*-4. *cis* 19:1 *n*-9. *cis* 20:1 *n*-15. *cis* 20:1 *n*-12. *cis* 20:1 *n*-9. *cis* 22:1 *n*-9. *cis* 24:1 *n*-9. Total PUFAs include: 18:2 *n*-6. 18:3 *n*-6. 18:3 *n*-3. 20:2 *n*-6. 20:3 *n*-6. 20:4 *n*-6. 20:3 *n*-3. 20:5 *n*-3. *trans* 22:2 *n*-7. *cis* 22:3 *n*-3/*cis* 22:4 *n*-5. 22:5 *n*-3. 22:6 *n*-3. Total *n*-3 include: 18:3 *n*-3. 20:3 *n*-3. 20:5 *n*-3. 22:5 *n*-3. 22:6 *n*-3. Total *n*-6 include: 18:2 *n*-6. 18:3 *n*-6. 20:3 *n*-6. 20:4 *n*-6; ^a^ Wilcoxon signed-rank test for matched data; ^b^ *r* Spearman’s correlation coefficient.

**Table 3:** Adjusted association between maternal/infant characteristics and maternal FAs percentage.

Dependent Variable	Independent Variable	Coeff.	95% CI		*p*
			Lower Bound	Upper Bound	
**14:0**	**Smoking (Ref.: no)**	0.375	0.203	0.547	***0.000***
	**Maternal age**	0.018	0.006	0.030	***0.003***
	**Education level (Ref.: low)**	−0.317	−0.503	−0.132	***0.001***
**16:0**	**Gestational age**	−0.435	−0.798	−0.072	***0.019***
***trans* 16:1 *n*-7**	**Offspring sex (Ref.: male)**	−0.134	−0.216	−0.052	***0.001***
**18:2, *n*-6 (LA)**	**Pre-pregnancy BMI**	−0.033	−0.066	−0.001	***0.044***
	**Maternal age**	−0.036	−0.064	−0.009	***0.009***
**18:3, *n*3 (ALA)**	**Education level (Ref.: low)**	−0.090	−0.138	−0.042	***0.000***
**20:5, *n*-3 (EPA)**	**Offspring sex (Ref.: male)**	−0.060	−0.103	−0.016	***0.008***
**22:5, *n*-3 (DPA)**	**Inadequate GWG**	0.637	0.271	1.002	***0.001***
	**Adequate GWG (ref.)**	-	-	-	***-***
	**Excessive GWG**	−0.078	−0.429	0.272	0.661
**22:6, *n*-3 (DHA)**	**Inadequate GWG**	0.337	−0.152	0.828	0.177
	**Adequate GWG (ref.)**	-	-	-	***-***
	**Excessive GWG**	−0.714	−1.200	−0.228	***0.004***
	**Pre-pregnancy BMI**	0.055	0.010	0.099	***0.016***
	**Gestational age**	0.343	0.183	0.503	***0.000***
**Total MUFAs**	**Pre-pregnancy BMI**	−0.112	−0.207	−0.017	***0.021***
**Total PUFAs**	**Gestational age**	0.662	0.075	1.248	***0.027***
**Total *n*-3**	**Gestational age**	0.254	0.020	0.488	***0.033***
**Total *n*-6**	**Maternal age**	−0.096	−0.177	−0.015	***0.021***

Multivariate quantile regressions between maternal erythrocyte FAs (dependent variable) and the independent co-variables: GWG, pre-pregnancy BMI, smoking, maternal age, educational level, offspring sex, gestational age, and parity. Continuous variables: pre-pregnancy BMI, maternal age, gestational age. Categorical variables: GWG (Ref.: adequate), smoking (Ref.: no), education level (Ref.: low), Offspring sex (Ref.: male), Parity (Ref.: 0). ALA, alpha-linolenic acid; BMI, body mass index = kg/m^2^; CI, confidence interval; DGLA, dihomo-gamma-linoleic acid; DHA, docosahexaenoic acid; DPA, docosapentaenoic acid; EPA, eicosapentaenoic acid; GWG, gestational weight gain; LA, linoleic acid; MUFAs, monounsaturated fatty acids; PUFAs, polyunsaturated fatty acids. Total PUFAs include: 18:2 *n*-6, 18:3 *n*-6, 18:3 *n*-3, 20:2 *n*-6, 20:3 *n*-6, 20:4 *n*-6, 20:3 *n*-3, 20:5 *n*-3, 22:2 *n*-7, *cis* 22:3 *n*-3/*cis* 22:4 *n*-5, 22:5 *n*-3, 22:6 *n*-3. Total MUFAs include: *cis* 12:1 *n*-1. 14:1 *n*-5. *cis* 15:1 *n*-1. *trans* 16:1 *n*-7. *cis* 16:1 *n*-7. *cis* 17:1 *n*-7. *trans* 18-1 *n*-9. *cis* 18:1 *n*-9. *trans* 18-1 *n*-7. *cis* 18:1 *n*-7. 18:1 *n*-5. *n*-4.*cis* 19:1 *n*-9. *cis* 20:1 *n*-15. *cis* 20:1 *n*-12. *cis* 20:1 *n*-9. *cis* 22:1 *n*-9. *cis* 24:1 *n*-9. Total *n*-3 include: 18:3 *n*-3, 20:3 *n*-3, 20:5 *n*-3, 22:5 *n*-3, 22:6 *n*-3. Total *n*-6 include: 18:2 *n*-6. 18:3 *n*-6. 20:3 *n*-6. 20:4 *n*-6.

**Table 4:** Adjusted association between maternal/foetal characteristics and foetal FAs percentage.

Dependent Variable	Independent Variable	Coeff.	95% CI		*p*
			Lower Bound	Upper Bound	
**14:0**	**Maternal 14:0**	0.779	0.727	0.831	***0.000***
	**Education level (Ref.: low)**	0.166	0.060	0.271	***0.002***
**16:0**	**Maternal 16:0**	0.654	0.569	0.738	***0.000***
**18:0**	**Maternal 18:0**	0.696	0.606	0.786	***0.000***
***trans* 16:1 *n*-7**	**Maternal 16:1t**	0.291	0.210	0.371	***0.000***
**18:2, *n*-6 (LA)**	**maternal 18:2, *n*-6**	0.111	0.078	0.143	***0.000***
**20:5, *n*-3 (EPA)**	**Maternal EPA**	0.591	0.541	0.641	***0.000***
**22:5, *n*-3 (DPA)**	**Maternal DPA**	0.482	0.454	0.510	***0.000***
**22:6, *n*-3 (DHA)**	**Maternal DHA**	0.590	0.520	0.661	***0.000***
	**Pre-pregnancy BMI**	−0.029	−0.055	−0.002	***0.036***
**20:4, *n*-6 (AA)**	**Maternal AA**	0.948	0.839	1.058	***0.000***
**20:3, *n*-6 (DGLA)**	**Maternal 20:3, *n*-6**	1.113	0.993	1.234	***0.000***
**Total SFAs**	**Maternal SFAs**	0.539	0.421	0.657	***0.000***
**Total MUFAs**	**Maternal MUFAs**	0.277	0.217	0.337	***0.000***
**Total PUFAs**	**Maternal PUFAs**	0.415	0.317	0.513	***0.000***
	**Pre-pregnancy BMI**	−0.172	−0.301	−0.043	***0.009***
**Total *n*-3**	**Maternal omega 3**	0.678	0.623	0.733	***0.000***
	**Smoking (Ref.: no)**	0.513	0.058	0.968	***0.027***
**Total *n*-6**	**Maternal omega 6**	0.697	0.618	0.776	***0.000***
	**Pre-pregnancy BMI**	−0.098	−0.177	−0.019	***0.015***
	**Smoking (Ref.: no)**	1.031	0.057	2.005	***0.038***

Multivariate quantile regressions between foetal erythrocyte fatty acids (dependent variable) and the independent co-variables: maternal correspondent fatty acid, GWG, pre-pregnancy BMI, smoking, maternal age, educational level, offspring sex, gestational age, parity. Continuous variables: pre-pregnancy BMI, maternal age, gestational age. Categorical variables: GWG (Ref.: adequate), smoking (Ref.: no), education level (Ref.: low), Offspring sex (Ref.: male), Parity (Ref.: 0). Significant results for *p* < 0.05. BMI, body mass index = kg/m^2^; AA, arachidonic acid; ALA, alpha-linolenic acid; CI, confidence interval; DGLA, dihomo-gamma-linolenic acid; DHA, docosahexaenoic acid; DPA, docosapentaenoic acid; EPA, eicosapentaenoic acid; FAs, fatty acid methyl ester; LA, linoleic acid; MUFAs, monounsaturated fatty acids; PUFAs, polyunsaturated fatty acids; SFAs, saturated fatty acids; UFAs, unsaturated fatty acid. Total SFAs include: 12:0. 13:0. 14:0. 15:0. 16:0. 17:0. 18:0. 19:0. 20:0. 22:0. 24:0. Total MUFAs include: *cis* 12:1 *n*-1. 14:1 *n*-5. *cis* 15:1 *n*-1. *trans* 16:1 *n*-7. *cis* 16:1 *n*-7. *cis* 17:1 *n*-7. *trans* 18-1 *n*-9. *cis* 18:1 *n*-9. *trans* 18-1 *n*-7. *cis* 18:1 *n*-7. 18:1 *n*-5. *n*-4. *cis* 19:1 *n*-9. *cis* 20:1 *n*-15. *cis* 20:1 *n*-12. *cis* 20:1 *n*-9. *cis* 22:1 *n*-9. *cis* 24:1 *n*-9. Total PUFAs include: 18:2 *n*-6. 18:3 *n*-6. 18:3 *n*-3. 20:2 *n*-6. 20:3 *n*-6. 20:4 *n*-6. 20:3 *n*-3. 20:5 *n*-3. *trans* 22:2 *n*-7. *cis* 22:3 *n*-3/*cis* 22:4 *n*-5. 22:5 *n*-3. 22:6 *n*-3. Total *n*-3 include: 18:3 *n*-3. 20:3 *n*-3. 20:5 *n*-3. 22:5 *n*-3. 22:6 *n*-3. Total *n*-6 include: 18:2 *n*-6. 18:3 *n*-6. 20:3 *n*-6. 20:4 *n*-6.

## Supplementary Materials

The following are available online at http://www.mdpi.com/2072-6643/8/6/368/s1, Figure S1: Maternal and foetal samples divided according to DHA threshold fixed at 3.4% of total fatty acids. Plots of DHA% in maternal and foetal erythrocyte membranes in mothers with DHA ≤ 3.4% (*n* = 247) and >3.4% (*n* = 188). Statistically significant differences were found between maternal and foetal percentages of DHA in both groups (*p* = 0.000 for both, Wilcoxon signed-rank test for matched data). Continuous lines represent median values of DHA. DHA ≤ 3.4%: maternal = 2.2 (1.4–2.7); foetal = 2.6 (1.9–3.4). DHA > 3.4%: maternal = 4.7 (4.1–5.6); foetal = 4.1 (3.2–5.3), Table S1: Concentration of FAs in maternal and foetal erythrocytes (*n* = 435), Table S2: Unadjusted association between maternal/infant characteristics and maternal FAs percentage, Table S3: Unadjusted association between maternal/infant characteristics and foetal FAs percentage.

## Abbreviations

The following abbreviations are used in this manuscript:

AA	Arachidonic Acid
ADA	American Diabetes Association
ALA	α-Linolenic Acid
BL	Body Length
BMI	Body Mass Index
BW	Body Weight
DGLA	Dihomogamma Linoleic Acid
DHA	Docosaexahenoic Acid
DPA	Docosapentaenoic Acid
EDTA	Ethylenediaminetetraacetic Acid
EFAs	Essential Fatty Acids
EPA	Ecosapentaenoic acid
FAME	Fatty Acid Methyl Esters
FAs	Fatty Acids
GDM	Gestational Diabetes Mellitus
GWG	Gestational Weight Gain
HC	Head Circumference
INeS	Italian Neonatal Study
IQR	Inter Quartile Range
LA	linoleic acid
LC-PUFAs	Long Chain Unsaturated Fatty Acids
MUFAs	Monounsaturated Fatty Acids
OPBG	*Ospedale Pediatrico Bambino Gesù*
PUFAs	Polyunsaturated Fatty Acids
SCH	*San Camillo Forlanini* Hospital
SD	Standard Deviation
SDS	Standard Deviation Score
SES	Socioeconomic Status
SFAs	Saturated Fatty Acids
T1D	Type 1 Diabetes
T2D	Type 2 Diabetes
UFAs	Unsaturated Fatty Acids
WHO	World Health Organization
