# Supplementary material for: Influence of Maternal Obesity and Gestational Weight Gain on Maternal and Foetal Lipid Profile
Source: Nutrients. 2016 Jun 15;8(6):368. doi: 10.3390/nu8060368 (PMC4924209; doi:10.3390/nu8060368)
Supplement: Supplementary file 1 [file nutrients-08-00368-s001.docx]

Supplementary Materials: Influence of Maternal Obesity and Gestational Weight Gain on Maternal and Foetal Lipid Profile

Giulia Cinelli, Marta Fabrizi, Lucilla Ravà, Marta Ciofi degli Atti, Pamela Vernocchi,
Cristina Vallone, Emanuela Pietrantoni, Rosalba Lanciotti, Fabrizio Signore and Melania Manco

**Figure S1.** Maternal and foetal samples divided according to DHA threshold fixed at 3.4% of total fatty acids. Plots of DHA% in maternal and foetal erythrocytes membranes in mothers with DHA ≤ 3.4% (*n* = 247) and >3.4% (*n* = 188). Statistically significant differences were found between maternal and foetal percentages of DHA in both groups (*p* = 0.000 for both, Wilcoxon signed-rank test for matched data). Continuous lines represent median values of DHA. DHA ≤ 3.4%: maternal = 2.2 (1.4–2.7); foetal = 2.6 (1.9–3.4). DHA > 3.4%: maternal = 4.7 (4.1–5.6); foetal = 4.1 (3.2–5.3).

**Table S1.** Concentration of FAs in maternal and foetal erythrocytes (*n* = 435).

| **Fatty Acids ^a^** | **Mean** | **Median** | **25° Perc.** | **75° Perc.** | **Mean** | **Median** | **25° Perc.** | **75° Perc.** | **Correlation Maternal *vs*. Fetal2** | | |
| --- | --- | --- | --- | --- | --- | --- | --- | --- | --- | --- | --- |
|  | **Maternal** | | | | **Foetal** | | | | ***p* ^a^** | ***r* ^b^** | ***p* ^b^** |
| **12:0** | 125.60 | 69.45 | 0.00 | 171.11 | 130.22 | 75.45 | 0.00 | 173.37 | 0.176 | 0.6 | ***0.000*** |
| **13:0** | 32.38 | 0.00 | 0.00 | 30.74 | 31.83 | 0.00 | 0.00 | 35.37 | 0.071 | 0.6 | ***0.000*** |
| **14:0** | 89.51 | 66.65 | 28.20 | 117.02 | 94.51 | 72.15 | 29.44 | 126.39 | 0.264 | 0.7 | ***0.000*** |
| **15:0** | 39.68 | 28.24 | 9.97 | 56.72 | 37.23 | 24.46 | 7.84 | 52.72 | 0.271 | 0.5 | ***0.000*** |
| **16:0** | 1724.95 | 1279.48 | 647.27 | 2346.92 | 1822.11 | 1343.13 | 682.80 | 2666.11 | ***0.036*** | 0.7 | ***0.000*** |
| **17:0** | 34.02 | 27.43 | 11.52 | 48.78 | 35.60 | 30.22 | 11.61 | 51.03 | 0.142 | 0.4 | ***0.000*** |
| **18:0** | 1167.92 | 885.92 | 471.00 | 1612.12 | 1307.11 | 969.85 | 520.85 | 1889.65 | ***0.000*** | 0.7 | ***0.000*** |
| **19:0** | 2.25 | 0.00 | 0.00 | 0.00 | 0.99 | 0.00 | 0.00 | 0.00 | 0.062 | 0.4 | ***0.000*** |
| **20:0** | 30.26 | 20.46 | 9.13 | 45.10 | 35.59 | 23.51 | 10.14 | 50.24 | ***0.000*** | 0.7 | ***0.000*** |
| **22:0** | 100.18 | 75.14 | 32.43 | 138.17 | 90.12 | 68.19 | 28.32 | 126.78 | ***0.001*** | 0.8 | ***0.000*** |
| **24:0** | 309.23 | 233.75 | 113.34 | 406.49 | 333.63 | 243.72 | 109.91 | 468.45 | ***0.043*** | 0.7 | ***0.000*** |
| ***cis* 12:1, *n*-1** | 96.69 | 39.23 | 0.00 | 143.13 | 106.24 | 35.03 | 0.00 | 150.00 | 0.105 | 0.8 | ***0.000*** |
| ***cis* 14:1, *n*-5** | 83.62 | 27.76 | 0.00 | 124.91 | 85.69 | 28.77 | 0.00 | 117.87 | 0.165 | 0.8 | ***0.000*** |
| ***cis* 15:1, *n*-1** | 15.86 | 0.00 | 0.00 | 8.02 | 8.62 | 0.00 | 0.00 | 8.00 | 0.268 | 0.6 | ***0.000*** |
| ***trans* 16:1, *n*-7** | 14.95 | 3.82 | 0.00 | 20.80 | 17.53 | 0.00 | 0.00 | 23.38 | 0.669 | 0.3 | ***0.000*** |
| ***cis* 16:1, *n*-7** | 42.08 | 27.94 | 0.00 | 64.55 | 34.31 | 14.52 | 0.00 | 54.32 | ***0.002*** | 0.2 | ***0.000*** |
| ***cis* 17:1, *n*-7** | 21.38 | 0.00 | 0.00 | 24.04 | 14.41 | 0.00 | 0.00 | 16.92 | ***0.000*** | 0.7 | ***0.000*** |
| ***trans* 18:1, *n*-9** | 17.56 | 0.00 | 0.00 | 0.00 | 21.77 | 0.00 | 0.00 | 0.00 | 0.157 | 0.4 | ***0.000*** |
| ***cis* 18:1, *n*-9** | 716.05 | 406.33 | 168.65 | 878.41 | 552.00 | 293.05 | 43.58 | 720.96 | ***0.000*** | 0.7 | ***0.000*** |
| ***trans* 18:1, *n*-7** | 328.25 | 0.00 | 0.00 | 258.10 | 254.72 | 0.00 | 0.00 | 314.06 | ***0.005*** | 0.7 | ***0.000*** |
| ***cis* 18:1, *n*-7** | 77.75 | 56.68 | 22.68 | 104.89 | 124.06 | 87.47 | 39.55 | 162.87 | ***0.000*** | 0.5 | ***0.000*** |
| ***cis* 19:1, *n*-9** | 9.63 | 0.00 | 0.00 | 15.65 | 9.48 | 0.00 | 0.00 | 13.30 | ***0.028*** | 0.5 | ***0.000*** |
| ***cis* 20:1, *n*-15** | 1.90 | 0.00 | 0.00 | 0.00 | 2.54 | 0.00 | 0.00 | 0.00 | 0.957 | 0.6 | ***0.000*** |
| ***cis* 20:1, *n*-12** | 14.34 | 0.00 | 0.00 | 16.70 | 10.44 | 3.61 | 0.00 | 12.04 | ***0.000*** | 0.6 | ***0.000*** |
| ***cis* 20:1, *n*-9** | 16.84 | 0.00 | 0.00 | 22.80 | 11.98 | 0.00 | 0.00 | 15.12 | ***0.000*** | 0.7 | ***0.000*** |
| ***cis* 22:1, *n*-9** | 6.31 | 0.00 | 0.00 | 7.12 | 8.84 | 0.00 | 0.00 | 9.75 | 0.181 | 0.5 | ***0.000*** |
| ***cis* 24:1, *n*-9** | 388.83 | 258.34 | 124.21 | 472.51 | 289.58 | 174.49 | 75.53 | 384.94 | ***0.000*** | 0.7 | ***0.000*** |
| **18:2, *n*-6 (LA)** | 482.13 | 334.85 | 150.25 | 647.38 | 244.67 | 163.56 | 74.17 | 342.96 | ***0.000*** | 0.6 | ***0.000*** |
| **18:3, *n*-6** | 14.14 | 6.49 | 0.00 | 22.91 | 13.08 | 3.85 | 0.00 | 20.25 | 0.114 | 0.5 | ***0.000*** |
| **18:3, *n*-3 (ALA)** | 6.81 | 0.00 | 0.00 | 10.47 | 2.94 | 0.00 | 0.00 | 0.00 | ***0.000*** | 0.1 | ***0.003*** |

**Table S1.** *Cont.*

| **20:2, *n*-6** | 20.00 | 9.13 | 0.00 | 26.16 | 48.57 | 29.62 | 12.63 | 66.57 | ***0.000*** | 0.1 | ***0.008*** |
| --- | --- | --- | --- | --- | --- | --- | --- | --- | --- | --- | --- |
| **20:3, *n*-6 (DGLA)** | 95.09 | 62.12 | 23.97 | 118.50 | 127.94 | 82.58 | 35.77 | 159.35 | ***0.000*** | 0.8 | ***0.000*** |
| **20:4, *n*-6 (AA)** | 644.01 | 430.65 | 113.56 | 813.53 | 807.81 | 546.12 | 203.14 | 1067.58 | ***0.000*** | 0.7 | ***0.000*** |
| **20:3, *n*-3** | 19.80 | 0.00 | 0.00 | 5.68 | 29.37 | 0.00 | 0.00 | 6.14 | 0.180 | 0.6 | ***0.000*** |
| **20:5, *n*-3 (EPA)** | 18.02 | 8.00 | 0.00 | 26.89 | 14. | 7.60 | 0.00 | 22.12 | ***0.004*** | 0.5 | ***0.000*** |
| ***trans* 22:2, *n*-7** | 50.81 | 35.32 | 10.61 | 75.54 | 26.44 | 14.50 | 0.00 | 39.49 | ***0.000*** | 0.4 | ***0.000*** |
| **22:5, *n*-3 (DPA)** | 70.38 | 16.74 | 0.00 | 86.94 | 46.91 | 0.00 | 0.00 | 0.00 | ***0.000*** | 0.8 | ***0.000*** |
| **22:6, *n*-3 (DHA)** | 332.54 | 198.71 | 90.18 | 455.77 | 353.60 | 218.94 | 94.06 | 537.16 | ***0.014*** | 0.8 | ***0.000*** |
| **Total FAa** | 9032.53 | 6696.11 | 3366.02 | 12,433.96 | 9803.01 | 6973.15 | 3770.91 | 14,100.28 | ***0.002*** | 0.8 | ***0.000*** |
| **Total SFAs** | 3665.00 | 2838.09 | 1429.33 | 5131.74 | 3911.12 | 3000.54 | 1555.32 | 5470.67 | ***0.006*** | 0.7 | ***0.000*** |
| **Total MUFAs** | 1876.26 | 1427.84 | 718.02 | 2516.64 | 1568.12 | 1153.45 | 617.54 | 2236.64 | ***0.000*** | 0.7 | ***0.000*** |
| **Total PUFAs** | 1884.11 | 1331.98 | 689.63 | 2384.50 | 1876.10 | 1300.68 | 668.69 | 2446.95 | 0.951 | 0.7 | ***0.000*** |
| **Total *n*-3** | 447.14 | 291.62 | 153.82 | 616.12 | 448.22 | 297.71 | 165.19 | 641.24 | 0.646 | 0.7 | ***0.000*** |
| **Total *n*-6** | 1235.27 | 879.93 | 404.02 | 1531.15 | 1194.06 | 799.85 | 375.72 | 1508.11 | 0.142 | 0.7 | ***0.000*** |

AA, arachidonic acid; ALA, alpha-linolenic acid; DGLA, dihomo-gamma-linolenic acid; DHA, docosahexaenoic acid; DPA, docosapentaenoic acid; EPA, eicosapentaenoic acid; FAs, fatty acid methyl ester; MUFAs, monounsaturated fatty acids; PUFAs, polyunsaturated fatty acids; SFAs, saturated fatty acids; UFAs, unsaurated fatty acid. Total SFAs include: 12:0. 13:0. 14:0. 15:0. 16:0. 17:0. 18:0. 19:0. 20:0. 22:0. 24:0. Total MUFAs include: *cis* 12:1 *n*-1. 14:1 *n*-5. *cis* 15:1 *n*-1. *trans* 16:1 *n*-7. *cis* 16:1 *n*-7. *cis* 17:1 *n*-7. *trans* 18-1 *n*-9. *cis* 18:1 *n*-9. *trans* 18-1 *n*-7. *cis* 18:1 *n*-7. 18:1 *n*-5. *n*-4.*cis* 19:1 *n*-9. *cis* 20:1 *n*-15. *cis* 20:1 *n*-12. *cis* 20:1 *n*-9. *cis* 22:1 *n*-9. *cis* 24:1 *n*-9. Total PUFAs include: 18:2 *n*-6. 18:3 *n*-6. 18:3 *n*-3. 20:2 *n*-6. 20:3 *n*-6. 20:4 *n*-6. 20:3 *n*-3. 20:5 *n*-3. *trans* 22:2 *n*-7. *cis* 22:3 *n*-3/*cis* 22:4 *n*-5. 22:5 *n*-3. 22:6 *n*-3. Total *n*-3 include: 18:3 *n*-3. 20:3 *n*-3. 20:5 *n*-3. 22:5 *n*-3. 22:6 *n*-3. Total *n*-6 include: 18:2 *n*-6. 18:3 *n*-6. 20:3 *n*-6. 20:4 *n*-6; ^a^ Wilcoxon signed-rank test for matched data; ^b^ *r*. Spearman’s correlation coefficient.

**Table S2.** Unadjusted association between maternal/infant characteristics and maternal FA percentage.

| **Dependent Variable** | **Independent Variable** | **Coeff.** | **95% CI** | | ***p*** |
| --- | --- | --- | --- | --- | --- |
|  |  |  | **Low** | **High** |  |
| **14:0** | **Smoking (Ref.: no)** | 0.352 | 0.160 | 0.544 | 0.000 |
|  | **Maternal age** | 0.017 | 0.002 | 0.033 | 0.028 |
|  | **Education level (Ref.: low)** | −0.397 | −0.624 | −0.171 | 0.001 |
| **16:0** | **Gestational age** | −0.435 | −0.798 | −0.072 | 0.019 |
| ***trans* 16:1, *n*-7** | **Offspring sex (Ref.: male)** | −0.134 | −0.216 | −0.052 | 0.001 |
|  | **Parity (Ref.: 0)** | 0.126 | 0.043 | 0.208 | 0.003 |
| **18:3, *n*-3 (ALA)** | **Education level (Ref.: low)** | −0.090 | −0.138 | −0.042 | 0.000 |
| **20:5, *n*-3 (EPA)** | **Offspring sex (Ref.: male)** | −0.060 | −0.103 | −0.016 | 0.008 |
| **22:5, *n*-3 (DPA)** | **Inadequate GWG** | 0.637 | 0.271 | 1.002 | 0.001 |
|  | **Maternal age** | −0.028 | −0.056 | −0.001 | 0.045 |
|  | **Gestational age** | 0.121 | 0.000 | 0.241 | 0.049 |
|  | **Parity (Ref.: 0)** | −0.458 | −0.760 | −0.157 | 0.003 |
| **22:6, *n*-3 (DHA)** | **Gestational age** | 0.220 | 0.023 | 0.417 | 0.029 |
| **Total MUFAs** | **Pre-pregnancy BMI** | −0.092 | −0.185 | 0.001 | 0.052 |
| **Total PUFAs** | **Inadequate GWG** | 1.827 | 0.146 | 3.508 | 0.033 |
|  | **Gestational age** | 0.662 | 0.075 | 1.248 | 0.027 |
| **Total *n*-3** | **Gestational age** | 0.254 | 0.020 | 0.488 | 0.033 |
| **Total *n*-6** | **Maternal age** | −0.096 | −0.177 | −0.015 | 0.021 |

Univariate quantile regressions between maternal erythrocyte fatty acids (dependent variable) and the independent variables: GWG, pre-pregnancy BMI, smoking, maternal age, educational level, offspring sex, gestational age, parity. Continuous variables: pre-pregnancy BMI, maternal age, gestational age. Categorical variables: GWG (Ref.: adequate), smoking (Ref.: no), education level (Ref.: low), offspring sex (Ref.: male), parity (Ref.: 0). Significant results for *p* < 0.05. Values are regression coefficients, *p*-values (*p*) and (95% CI); BMI, body mass index = kg/m^2^; CI, confidence interval; ALA, α-linolenic acid; DHA, docosaexahenoic acid; DPA, docosapentaenoic acid; EPA, eicosapentaenoic acid; FAs, fatty acids; GWG, gestational weight gain; MUFAs, monounsaturated fatty acids; PUFAs, polyunsaturated fatty acids. Total MUFAs include: *cis* 12:1 *n*-1, 14:1 *n*-5, *cis* 15:1 *n*-1, *trans* 16:1 *n*-7, *cis* 16:1 *n*-7, *cis* 17:1 *n*-7, *trans* 18-1 *n*-9, *cis* 18:1 *n*-9, *trans* 18-1 *n*-7, *cis* 18:1 *n*-7, *cis* 18:1 *n*-5, *n*-4, *cis* 19:1 *n*-9, *cis* 20:1 *n*-15, *cis* 20:1 *n*-12, *cis* 20:1 *n*-9, *cis* 22:1 *n*-9, cis 24:1 *n*-9. Total PUFAs include: 18:2 *n*-6, 18:3 *n*-6, 18:3 *n*-3, 20:2 *n*-6, 20:3 *n*-6, 20:4 *n*-6, 20:3 *n*-3, 20:5 *n*-3, 22:2 *n*-7, *cis* 22:3 *n*-3/*cis* 22:4 *n*-5, 22:5 *n*-3, 22:6 *n*-3. Total *n*-6 include: 18:2 *n*-6, 18:3 *n*-6, 20:3 *n*-6, 20:4 *n*-6. Total *n*-3 include: 18:3 *n*-3, 20:3 *n*-3, 20:5 *n*-3, 22:5 *n*-3, 22:6 *n*-3. Total *n*-6 include: 18:2 *n*-6. 18:3 *n*-6. 20:3 *n*-6. 20:4 *n*-6.

**Table S3.** Unadjusted association between maternal/infant characteristics and foetal FAs percentage.

| **Dependent Variable** | **Independent Variable** | **Coeff.** | **95% CI** | | ***p*** |
| --- | --- | --- | --- | --- | --- |
|  |  |  | **Low** | **High** |  |
| **Total MUFAs** | **Parity (Ref.: 0)** | −0.875 | −1.516 | −0.234 | 0.008 |
| **Total PUFAs** | **Pre-pregnancy BMI** | −0.142 | −0.261 | −0.023 | 0.019 |
|  | **Smoking (Ref.: no)** | 1731 | 0.150 | 3.311 | 0.032 |
| **Total *n*-6** | **Education level (Ref.: low)** | −1.250 | −2.395 | −0.105 | 0.032 |
| **14:0** | **Smoking (Ref.: no)** | 0.427 | 0.212 | 0.642 | 0.000 |
| **18:0** | **Pre-pregnancy BMI** | −0.076 | −0.150 | −0.002 | 0.044 |
| **20:5 *n*-3 (EPA)** | **Smoking (Ref.: no)** | −0.047 | −0.093 | −0.001 | 0.044 |
| **22:6 *n*-3 (DPA)** | **Inadequate GWG** | 0.334 | 0.146 | 0.522 | 0.001 |
|  | **Education level (Ref.: low)** | −0.292 | −0.517 | −0.067 | 0.011 |
|  | **Parity (Ref.: 0)** | −0.202 | −0.353 | −0.052 | 0.008 |

Univariate quantile regressions between foetal erythrocytes fatty acids (dependent variable) and the independent variables: maternal correspondent fatty acid, categorised GWG, pre-pregnancy BMI, smoking, maternal age, educational level, offspring sex, gestational age, parity. Continuous variables: pre-pregnancy BMI, maternal age, gestational age. Categorical variables: GWG (Ref.: adequate), smoking (Ref.: no), education level (Ref.: low), offspring sex (Ref.: male), parity (Ref.: 0). Significant results for *p* < 0.05. Values are regression coefficients, *p*-values (*p*) and (95% CI); BMI, body mass index = kg/m^2^; CI, confidence interval; DPA, docosapentaenoic acid; EPA, eicosapentaenoic acid; FAs, fatty acids; GWG, gestational weight gain; MUFAs, monounsaturated fatty acids; PUFAs, polyunsaturated fatty acids. Total MUFAs include: *cis* 12:1 *n*-1, 14:1 *n*-5, *cis* 15:1 *n*-1, *trans* 16:1 *n*-7, *cis* 16:1 *n*-7, *cis* 17:1 *n*-7, *trans* 18-1 *n*-9, *cis* 18:1 *n*-9, *trans* 18-1 *n*-7, *cis* 18:1 *n*-7, *cis* 18:1 *n*-5, *n*-4, *cis* 19:1 *n*-9, *cis* 20:1 *n*-15, *cis* 20:1 *n*-12, *cis* 20:1 *n*-9, *cis* 22:1 *n*-9, *cis* 24:1 *n*-9. Total PUFAs include: 18:2 *n*-6, 18:3 *n*-6, 18:3 *n*-3, 20:2 *n*-6, 20:3 *n*-6, 20:4 *n*-6, 20:3 *n*-3, 20:5 *n*-3, 22:2 *n*-7, *cis* 22:3 *n*-3/*cis* 22:4 *n*-5, 22:5 *n*-3, 22:6 *n*-3. Total *n*-6 include: 18:2 *n*-6, 18:3 *n*-6, 20:3 *n*-6, 20:4 *n*-6.
